# COVID‐19 cardiac arrest due to Prinzmetal's angina in a previously normal heart

**DOI:** 10.1002/ccr3.4205

**Published:** 2021-06-24

**Authors:** Melinda Wang, Andrew Talon, Mehrdad Saririan

**Affiliations:** ^1^ Department of Internal Medicine St. Joseph's Hospital and Medical Center Valleywise Health Medical Center Creighton University School of Medicine Phoenix Campus Phoenix AZ USA; ^2^ Department of Cardiology, Interventional Cardiology District Medical Group Creighton University School of Medicine University of Arizona Phoenix AZ USA

**Keywords:** acute coronary syndrome, atypical presentation, coronary vasospasm, COVID‐19, PEA, Prinzmetal's angina, ST‐segment elevation

## Abstract

In COVID‐19 patients who develop sudden ST elevations it is necessary to consider cardiac causes other than myocardial infarction, such as coronary vasospasm.

## Graphical abstract

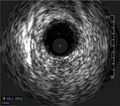

## 1 INTRODUCTION

There has been a compelling body of evidence of COVID‐19 causing cardiac complications such as myocarditis and increased risk of coronary thrombosis. Acute coronary syndrome due to coronary vasospasm is an unusual occurrence. We report a case of a 62‐year‐old man with severe respiratory syndrome coronavirus 2 (SARS‐CoV‐2) who presented with dyspnea. His hospital course was complicated by a pulseless electrical activity arrest. A coronary angiogram was significant for multifocal stenosis in the left anterior descending coronary artery that resolved with intracoronary nitroglycerin. Treatment modalities for ACS secondary to coronary vasospasm include nitrates and calcium channel blockers in hemodynamically stable patients.

Cardiac complications have been found to be prevalent in up to 30% of severe respiratory syndrome coronavirus 2 (SARS‐CoV‐2) patients.^1^ Although myocarditis has been the presumptive culprit of myocardial injury in the majority of cases, one‐third of SARS‐CoV‐2 patients showing ST‐segment elevation on the ECG have been found to have angiographically normal coronary arteries. This event suggests that another mechanism may be due to myocardial injury.^2^ We present a case of an acute coronary syndrome due to coronary vasospasm, rather than thrombosis, in a SARS‐CoV‐2 patient.

## 2 CASE DESCRIPTION

**FIGURE 1:**
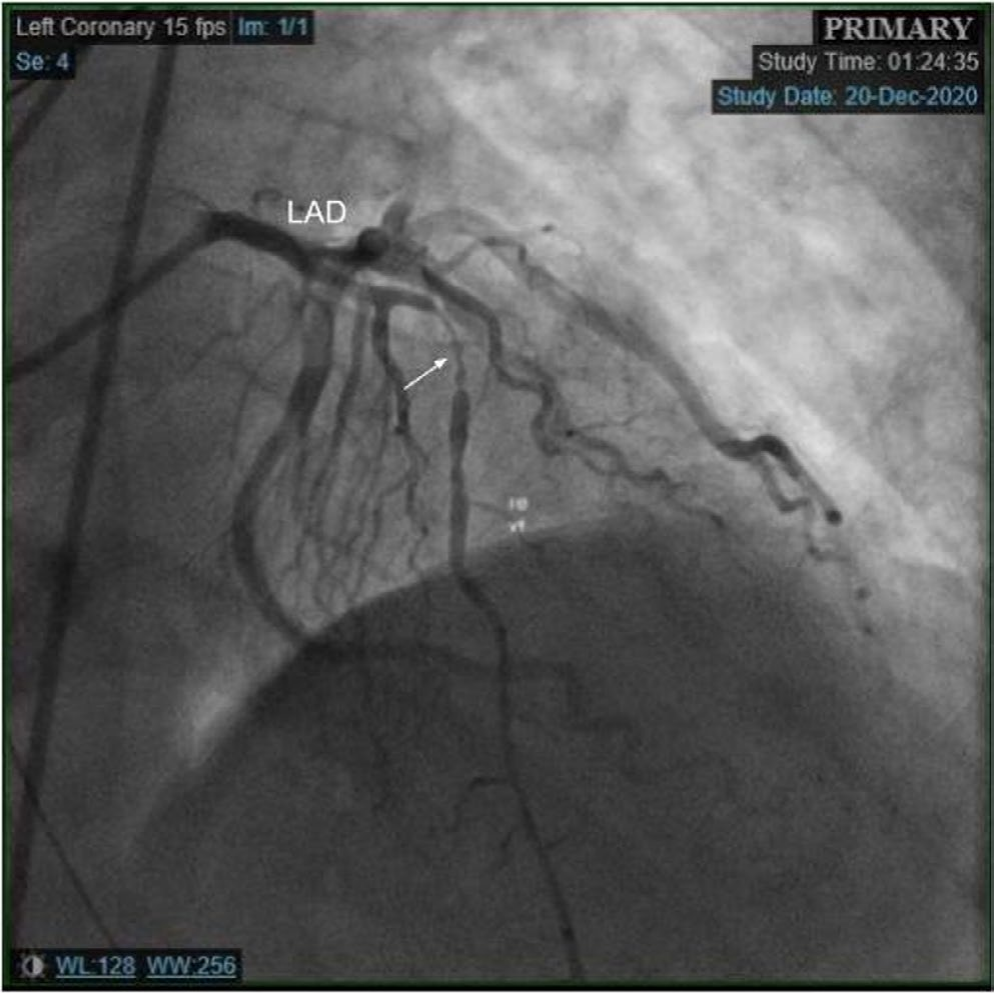
Electrocardiogram (ECG) showing ST elevation >0.1 mV in leads V2 through V6, suggesting ST‐segment elevation myocardial infarcti

**FIGURE 2:**
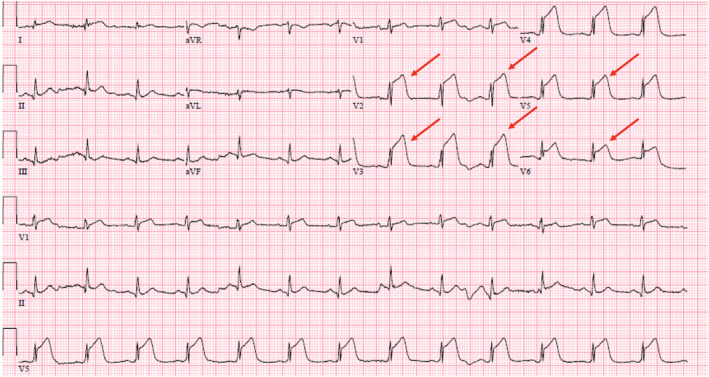
Left heart catheterization preintracoronary nitroglycerin injection illustrating multiple stenotic lesions in the left anterior descending artery suspected as coronary vasospasms. LAD—Left anterior descending artery

**FIGURE 3:**
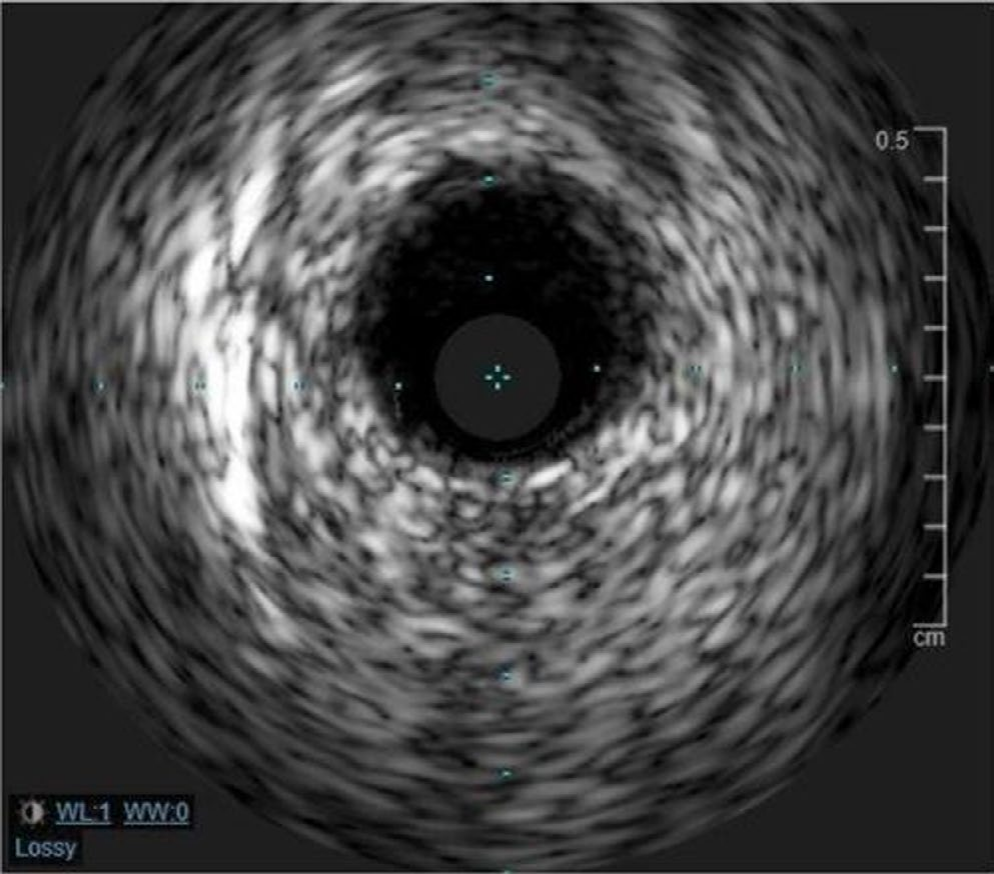
Left heart catheterization postintracoronary nitroglycerin injection and resolution of the stenotic lesions in the left anterior descending artery. LAD—Left anterior descending artery

**FIGURE 4:**
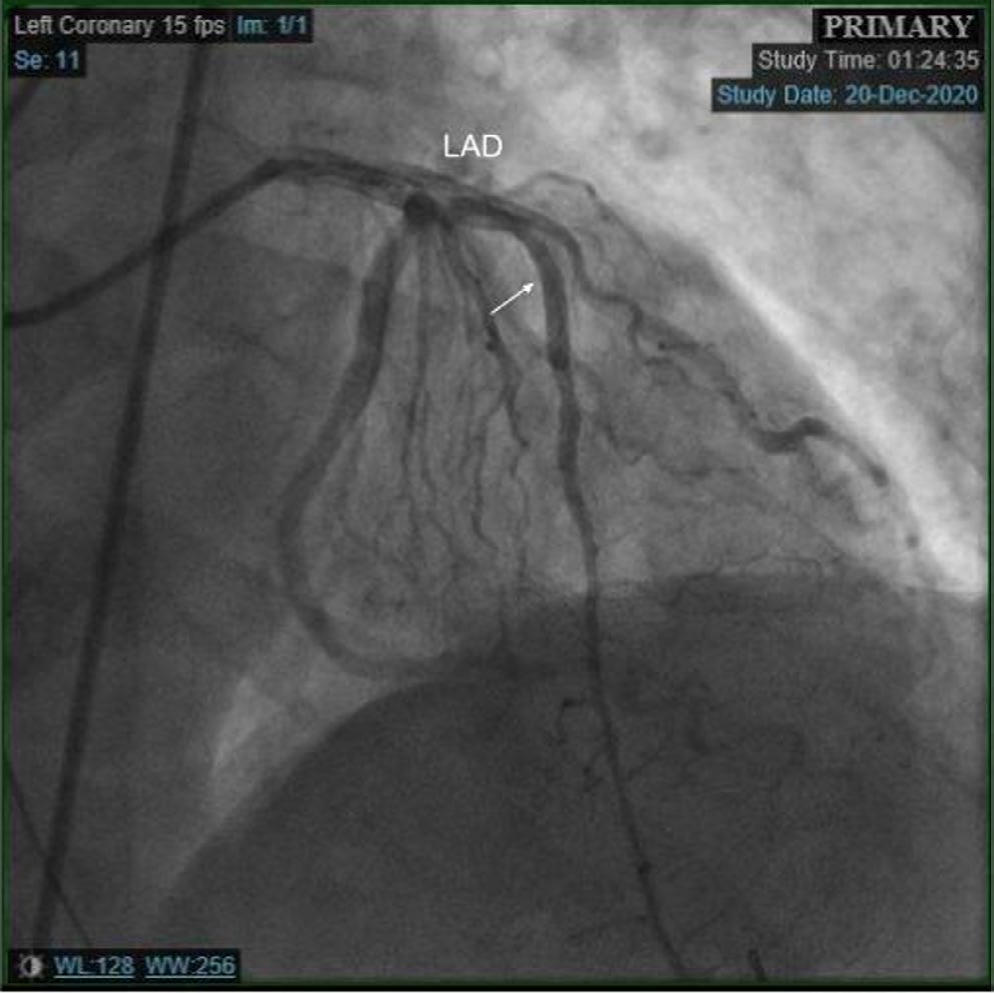
Intravascular ultrasound of the left anterior descending coronary artery without signs of atherosclerotic significant disease or lesions

A 62‐year‐old man diagnosed with COVID‐19 pneumonia presented 8 days after his positive COVID test with a chief complaint of worsening dyspnea. He had a prior history of bacterial pneumonia requiring hospitalizations and hypertension. He did not have any cardiac history. On presentation in the ED, the patient was hypoxic with 76% oxygen saturation on room air and required 15 L of oxygen/min through a nonrebreather mask to maintain his saturation levels above 90%. Computed tomography (CT) of the chest was negative for pulmonary embolism. He was treated with dexamethasone 6 mg daily (10‐day course), therapeutic dose anticoagulation (enoxaparin 1 mg/kg), and oxygen. The patient's daily complaints were dyspnea and cough, and he denied chest pain. On day 3 of his hospitalization, the patient's oxygen saturations decreased and the respiratory therapist was called to his room to increase his oxygen requirement. Before his oxygen could be maximized, he had cardiac arrest with pulseless electrical activity (PEA). Following resuscitation efforts, the patient was intubated and transferred to the intensive care unit. The patient's electrocardiogram was consistent with anterior lead ST‐segment elevations (Figure 1). His initial troponin I was 0.012 ng/mL. He was started on aspirin, high‐intensity statin, ticagrelor, and low intensity heparin infusion. Urgent coronary angiography revealed multifocal stenosis in the left anterior descending coronary artery (LAD), with myocardial infarction flow 3 (Figure 2). He otherwise had angiographically normal coronary vessels without signs of plaque erosion or rupture. The lesions in the LAD were treated with intracoronary nitroglycerin for suspected coronary spasm, revealing complete resolution of the stenotic segment with normal angiographic appearance of the vessel (Figure 3). Intravascular ultrasound was also performed revealing no significant atherosclerotic disease, remodeling, dissection, or thrombus (Figure 4). The patient's condition did not improve, and he was eventually placed on vasopressor support. He soon developed mixed cardiogenic and septic shock with multiorgan system failure. Laboratory examinations revealed D‐dimer of 1119 ng/mL. Inflammatory markers including erythrocyte sedimentation rate, ferritin, and C‐reactive protein (CRP) were not evaluated. Owing to the patient's need for ongoing vasopressor support, he was unable to be started on nitrates or calcium channel blockers. The patient ultimately passed away.

## 3 DISCUSSION

We diagnosed our patient with coronary vasospasm given the visible resolution of stenosis after intracoronary nitroglycerin. Our workup did not reveal any evidence of coronary artery disease or structural heart disease (Figure 4). A thromboembolic phenomenon would also be unlikely since the patient was receiving therapeutic anticoagulation throughout his hospitalization. Treatment modalities for coronary vasospasm after diagnosis include nitrates or calcium blockers. However, our patient was not a candidate for treatment due to his hemodynamic instability.

Coronary vasospasm is defined as a transient abnormal contraction of the muscle layer of an epicardial coronary artery resulting in myocardial ischemia.^1^ Previous cases of ST‐segment elevation have shown normal coronary arteries on angiography.^2^ In these cases, transient coronary vasospasm should be considered as a cause of the ST‐elevation changes seen in COVID‐19 patients. It is likely that coronary vasospasm is more prevalent than currently reported in COVID‐19. However, most patients do not have the vasospasm captured on cardiac catheterization. Although the pathogenesis is unclear, we postulate that the severe inflammatory response due to COVID‐19 may contribute to endothelial dysfunction and provoking a pulseless electrical activity arrest secondary to coronary vasospasm. Our theory is supported by prior studies reporting that the release of inflammatory mediators during hypersensitivity and anaphylactic reactions have been implicated in inducing coronary vasospasm.^3^ Hyperventilation is also another cause of coronary vasospasm and should be considered in COVID‐19 patients due to both respiratory distress and anxiety.^4^. Once coronary vasospasm is included as a differential for ST elevation in COVID patients, prophylactic nitrates or calcium channel blockers should be considered in order to prevent further demise.

## 4 CONCLUSION

COVID‐19 causes complications in many body systems and can be a difficult disease to navigate; thus, it is important to recognize atypical presentations. Our case highlighted the rare occurrence of coronary vasospasm associated with COVID‐19 in an otherwise normal heart that had no evidence of thrombosis or structural disease. It is important to recognize rare causes of cardiac complications that may at first appear to be a ST‐elevation MI. In COVID‐19 patients with sudden ST elevations, it may be also worthwhile to consider treating prophylactically for coronary vasospasm.

## CONFLICT OF INTEREST

None declared.

## AUTHOR CONTRIBUTIONS

MW, AT, MS: contributed to the analysis of the results and to the writing of the manuscript.

## CONSENT STATEMENT

A written informed consent was obtained from the patient for publication of identifying data.

## ETHICAL APPROVAL

Ethics approval to report this case was obtained from Valleywise Health Medical Center Institutional Review Board (IRB). The Valleywise Health IRB determined that a single case report, containing only deidentified data, does not produce generalizable knowledge, nor is it an investigation of an FDA regulated product. A case report is considered to be an educational activity, and extent from IRB reviewed based on the Code of Federal Regulations (CFRs) Title 45, Part 46.

## Data Availability

The authors confirm that the data supporting the findings of this research are available within the article.
